# Genes Encoding Cucumber Full-Size ABCG Proteins Show Different Responses to Plant Growth Regulators and Sclareolide

**DOI:** 10.1007/s11105-015-0956-9

**Published:** 2015-11-14

**Authors:** Adam Rajsz, Anna Warzybok, Magdalena Migocka

**Affiliations:** Department of Plant Molecular Physiology, University of Wroclaw, Institute of Experimental Biology, Kanonia 6/8, 50-328 Wrocław, Poland

**Keywords:** ATP-binding cassette, subfamily G (ABCG family)/pleiotropic drug resistance protein family (PDR), Phytohormones, Plant growth regulators, Sclareolide, Gene expression, Cucumber

## Abstract

Full-size members of the ABCG (ATP-binding cassette, subfamily G) subfamily of ABC transporters have been found only in plants and fungi. The plant genes encoding full-size ABCGs identified so far appeared to be differentially regulated under various environmental constraints, plant growth regulators, and microbial elicitors, indicating a broad functional role of these proteins in plant responses to abiotic and biotic stress. Nevertheless, the structure and physiological function of full-size ABCGs in many plant species are still unknown. We have recently identified 16 genes encoding full-size ABCG proteins in cucumber and found that the transcripts of two of them, *CsABCG36* (*CsPDR8*) and *CsABCG40* (*CsPDR12*), are most abundant in roots and are significantly affected by phytohormones and auxin herbicide. In this study, we analyzed the structure and phylogeny of all the full-size cucumber ABCG transporters and studied the organ expression profiles of the remaining 14 *CsABCG* genes. In addition, we investigated the effect of different plant growth regulators and the diterpene sclareolide on *CsABCG* expression in cucumber roots. Until now, the full-size plant ABCG transporters have been grouped into five different clusters. The new phylogenetic analysis of full-size ABCGs from model plants and cucumber clustered these proteins into six different subgroups. Interestingly, the expression profiles of cucumber *ABCG* genes assigned to the same clusters were not correlated, suggesting functional diversification or different regulatory mechanisms of the full-size cucumber ABCG proteins.

**Electronic supplementary material:**

The online version of this article (doi:10.1007/s11105-015-0956-9) contains supplementary material, which is available to authorized users.

## Introduction

ABC proteins are ubiquitous transporters mediating the translocation of a wide range of structurally unrelated molecules across biological membranes (Higgins 1992). They share highly conserved amino acid sequence domains designated as nucleotide binding domains (NBDs) and more variable transmembrane domains (TMDs). Each NBD contains three characteristic motifs of Walker A [GX_4_GK(ST)], Walker B boxes [(RK)X_3_GX_3_L (hydrophobic)_3_D] and an ABC signature [(LIVMFY)S(SG)GX_3_(RKA)(LIVMYA)X(LIVFM)(AG)], which is located between two Walker boxes (Bairoch 1992; Walker et al. 1982). Based on the modular organization of NBDs and TMDs, ABC proteins are classified as half-size transporters (composed of a single copy of NBDs and TMDs) or full-size transporters (composed of a minimum of two NBD and two TMDs). Since the genomic sequences of different plant species have been continuously released, the information on the organization and structure of plant ABC proteins has been rapidly growing. As a consequence, the inventories of plant ABC proteins are now available for the vascular plants *Arabidopsis thaliana*, *Oryza sativa*, *Lotus japonicus*, *Vitis vinifera*, *Solanum tuberosum*, and *Solanum lycopersicon* as well as for the green alga *Volvox carteri* (Garcia et al. 2004; Martinoia et al. 2002; Sanchez-Fernandez et al. 2001; Sugiyama et al. 2006; Çakır and Kılıçkaya 2013; Andolfo et al. 2015). Nevertheless, still little is known about homologous transporters in other plant families. Approximately 130 genes encoding putative ABC transporters have been identified in the *A. thaliana* genome (Martinoia et al. 2002; Sanchez-Fernandez et al. 2001; Schulz and Kolukisaoglu 2006). Fifty-four of these genes encode full-size transporters that fall into three subfamilies: ABCB (previously designated as multidrug resistance proteins, MDR), ABCC (previously designated as MDR-associated proteins, MRPs), and ABCG (comprising full-size pleiotropic drug resistance PDR proteins and half-size white-brown complex WBC homologs) (Crouzet et al. 2006; Sanchez-Fernandez et al. 2001; Theodoulou 2000; Trombik et al. 2008; van den Brule and Smart 2002; Verrier et al. 2008). Among these, the full-size members of the ABCG subfamily are particularly interesting, since they have been identified only in plants and fungi.

The genomes of two model plants, *A. thaliana* and *O. sativa*, contain 15 and 23 genes encoding full-size members of the ABCG subfamily, respectively (Sanchez-Fernandez et al. 2001; Crouzet et al. 2006). Similar number of genes encoding the full-size ABCGs has been identified in *Solanum lycopersicon* (23 genes) and *Solanum tuberosum* (25 genes) genomes, whereas in *Vitis vinifera* the PDR subfamily is encoded by 33 ORFs (Çakır and Kılıçkaya 2013; Andolfo et al. 2015). Nevertheless, the physiological function, substrate specificity, or regulatory mechanisms of most of these proteins in plants still remain to be established. The available data clearly suggest a significant role of plant full-size ABCG transporters in the resistance to pathogens and abiotic stresses, in the response to growth regulators, and in cellular detoxification. Namely, *Arabidopsis* AtABCG40 (AtPDR12) and its structural homologs in *Nicotiana plumbaginifolia* and *Spirodela polyrrhiza* appear to contribute to the efflux of sclareol, an antifungal diterpene (Campbell et al. 2003; Jasinski et al. 2001; Stukkens et al. 2005; van den Brule et al. 2002). Orthologous *Arabidopsis* protein AtABCG36 (AtPDR8) is involved in plant defense against pathogen infection (Kobae et al. 2006; Stein et al. 2006). In addition, the newly discovered *Triticum aestivum* TaPDR1 transporter was shown to be induced by the pathogenic fungus *Fusarium graminearum* to confer wheat resistance to deoxynivalenol (DON)—the toxic fungal compound (Yi et al. 2009). Recent studies on the full-size ABCGs from *Petunia hybrida* and *Medicago truncatula* have clearly confirmed that members of this subfamily in plants are associated with plant interaction with pathogenic or symbiotic microorganisms (Banasiak et al. 2013) or mycorrhizae formation (Kretzschmar et al. 2012; Zhang et al. 2010).

The full-size members of the ABCG subfamily have also been implicated in the plant response to abiotic stresses through the transmembrane transport of toxic substances or stress-related regulatory compounds. Both AtABCG36 and AtABCG40 contribute to Cd detoxification and Pb resistance, respectively (Kim et al. 2007; Lee et al. 2005). In addition, AtABCG40 mediates the cellular uptake of phytohormone abscisic acid (ABA), involved in plant response to drought conditions (Kang et al. 2010). Beside AtABCG40, also AtABCG37 (AtPDR9) contributes to the translocation of regulatory compounds across biological membranes. It is capable of transport of phenoxyalkanoic acid family herbicides (e.g., 2,4-dichlorophenoxyacetic acid (2,4-D)) as well as the inhibitor of polar auxin transport naphthylphthalamic acid (NPA) out of plant cells (Ito and Gray 2006; Ruzicka et al. 2010). In contrast, AtABCG39 (AtPDR11), another full-size ABCG protein from *A. thaliana*, has been shown to act as an importer in cellular uptake of the non-selective herbicide paraquat (Xi et al. 2012). Moreover, the full-size ABCGs from plants has recently been shown to be essential for the proper formation of cuticle, a multilayered structure composed of polyester, cutin, and wax that functions as a transpiration barrier required for the water conservation in plant tissues and participates in plant-microbe interaction (Bessire et al. 2011; Chen et al. 2011; Yeats and Rose 2013). It has been demonstrated that the disruption of genes encoding the AtABCG32 and HvABCG31 proteins in *A. thaliana* and *Hordeum vulgare*, respectively, leads to the reduced deposition and increased permeability of cuticle on leaves and petals and thus, markedly diminishes the ability of plants to retain water in their tissues (Bessire et al. 2011; Chen et al. 2011).

A growing number of reports demonstrate that the full-size ABCG proteins in plants not only transport a range of different regulatory and structural compounds, but are also regulated by hormonal signals at the level of transcription, implicating an essential role of this family in hormone-mediated plant reactions to environmental or developmental stimuli. Expression of the *NtPDR3* gene from *Nicotiana tabacum* is highly induced by methyl jasmonate and salicylic acid—phytohormonal mediators of the plant response to abiotic and biotic stresses (Ducos et al. 2005). Abscisic acid significantly up-regulates the gene encoding SpTUR2, an ABCG33*-*like transporter from *Spirodela polyrrhiza*, whereas cytokinins and jasmonic acid markedly increase the accumulation of the *OsABCG36* (*OsPDR9*) transcript in *O. sativa* (Moons 2003; Smart and Fleming 1996). Furthermore, the expression of at least 12 of 23 genes encoding the full-size ABCG protein in rice is significantly affected by phytohormones such as ABA, auxins, jasmonic acid, cytokinins, and by other plant growth regulators (Moons 2003).

The lack of full genetic resources for most plant species is a serious limitation for obtaining a full view of the biological function of the ABCG family in plants. However, the current sequencing projects focused on non-model species without an available reference genome should provide new tools for genetic and functional studies of newly identified ABCGs. The sequencing of the cucumber genome revealed the presence of 16 genes encoding putative full-size ABCG proteins in this plant. We have recently shown that two of them, *CsABCG36* (*CsPDR8*) and *CsABCG40* (*CsPDR12*), are predominantly expressed in roots and transcriptionally regulated by phytohormones (ABA, ethylene, jasmonates, salicylic acid, cytokinins) and the herbicide 2,4-D (Migocka et al. 2012). In this study, we analyzed the structure and phylogeny of all the full-size CsABCG transporters and studied the organ expression patterns of 14 unstudied *CsABCG* genes in cucumber. In addition, we investigated the effect of different plant growth regulators and the analog of the antifungal diterpene sclareol on root-expressed *CsABCG* genes with regard to the *cis* elements found in their putative promoter sequences.

## Materials and Methods

### Plant Material

Cucumber plants (*Cucumis sativus*, var. *Krak*) were grown hydroponically under a 16-h photoperiod at 25 °C during the day and 22 °C during the night in 3-fold diluted Hogland solution, pH 6.0, as described earlier (Migocka and Papierniak 2011). The nutrient solution was filter-sterilized and exchanged twice a week. For organ expression analyses, particular organs of 1-week-old seedlings (roots, hypocotyls, cotyledons, leaves) or 8-week-old plants (roots, stems, leaves, petioles, tendrils, flowers, and fruits) were collected, immediately frozen in liquid nitrogen, and stored at −80 °C until use. For the analysis of hormonal responsiveness, 1-week-old seedlings were transferred for 4, 8, or 12 h onto the fresh nutrient solutions containing different concentrations of ABA, indole-3-acetic acid (IAA), 2,4-D, kinetin, salicylic acid (SA), gibberellic acid (GA_3_), 1-aminocyclopropane-1-carboxylic acid (ACC), jasmonic acid (JA), or the analog of antifungal diterpene sclareol—sclareolide. The final concentrations of phytohormones and growth regulators are given in the figure captions. The concentrations of all compounds were chosen based on previous reports (Jasinski et al. 2001; Migocka et al. 2012) and preliminary experiments. After treatment, the root seedlings were thoroughly washed, frozen in liquid nitrogen, and stored at −80 °C until use. For each analysis, four organ samples of 100 mg from four different cucumber plants were taken for RNA extraction.

### Preparation of cDNA for RT-PCR and Real-Time PCR

The harvested and frozen samples were ground to a fine powder under liquid nitrogen. Total RNA was extracted using TRI Reagent (Sigma-Aldrich) according to the manufacturer’s instructions. The RNA integrity was checked by electrophoresis, whereas RNA quality and quantity were determined spectrophotometrically (NanoDrop). Only the samples with A260/A280 and A260/80 values in the range of 1.9–2.0 or 1.8–2.2 were used for further analysis. RNA samples were treated with DNase I (Fermentas) to remove contaminating genomic DNA. cDNA was synthesized from 2000 ng of RNA by priming with random primers using the High-Capacity cDNA Reverse Transcription Kit (Applied Biosystems) according to the manufacturer’s instructions. The primers specific for each PDR gene were carefully designed using LightCycler Probe Design Software 2.0 (Roche) (Supplementary Table 1).

### RT-PCR

In order to determine the organ expression profile of *CsPDRs*, RT-PCR was performed using 1 U of Marathon polymerase (A&A Biotechnology), 1× Marathon buffer, 0.5 μM dNTP mix, 1 μl of 10 μM forward primers, 1 μl of 10 μM reverse primers, and 1 μl of cDNA prepared from different cucumber organs in a total reaction volume of 20 μl. The reaction conditions were as follows: initial denaturation for 2 min at 94 °C, followed by 30–34 cycles of 30 s denaturation at 94 °C, annealing for 30 s at the appropriate annealing temperature, and extension for 1 min at 68 °C (Supplementary Table 2). The program ended with a 10-min-long final extension at 68 °C. Genes encoding 18S RNA (accession AF206894 in GenBank, primers forward: 5′-TACCACATCCAAGGAAGGCAGCA-3′ and reverse: 5′-TGGAATTACCGCGGCTGCTGGCA-3′) and Clathrine Adaptor Complex Subunit (CACS, accession GW881874 in GenBank, forward primer: 5′-TGGGAAGATTCTTATGAAGTGC-3′ and reverse primer: CTCGTCAAATTTACACATTGGT) were used as reference controls. The PCR products were electrophoresed in a 1.8 % agarose gel, stained with ethidium bromide, and photographed.

### Quantitative Real-Time PCR

Quantitative RT-PCR was performed with LightCycler 480 System Real-Time PCR (Roche). The reaction mixture contained 2 μl of cDNA diluted eight times, 10 μl of 2× SYBR Master Mix B (A&A Biotechnology), 2 μl of 10 μM forward primer, and 2 μl of 10 μM reverse primer in a 20 μl solution. Amplifications were carried out in 94-well plates (Roche) after pre-incubation at 94 °C for 30 s, followed by 45 cycles of denaturation at 94 °C for 10 s, annealing at 55 °C for 10 s, and extension at 68 °C for 12 s, with final melting at 70 °C for 30 s. *CsPDRs* expression data were normalized to the expression of two reference genes encoding CACS and TIP41 (tonoplast intrinsic protein), which proved to be highly stable under plant growth regulator treatments (Migocka and Papierniak 2011). A negative control without a cDNA template was included in the same PCR run for each primer pair. To confirm the specificity of amplification, melting curve analysis was performed allowing identification of putative unspecific PCR products (e.g., primer dimers, reaction mix contamination). Real-time PCR efficiencies were in the range 90–105 % as calculated from the slopes generated in LightCycler software. For each of the three independent RNA extractions, measurements of gene expression were obtained in triplicate.

### Database Search and Sequence Analyses

**Fig. 1:**
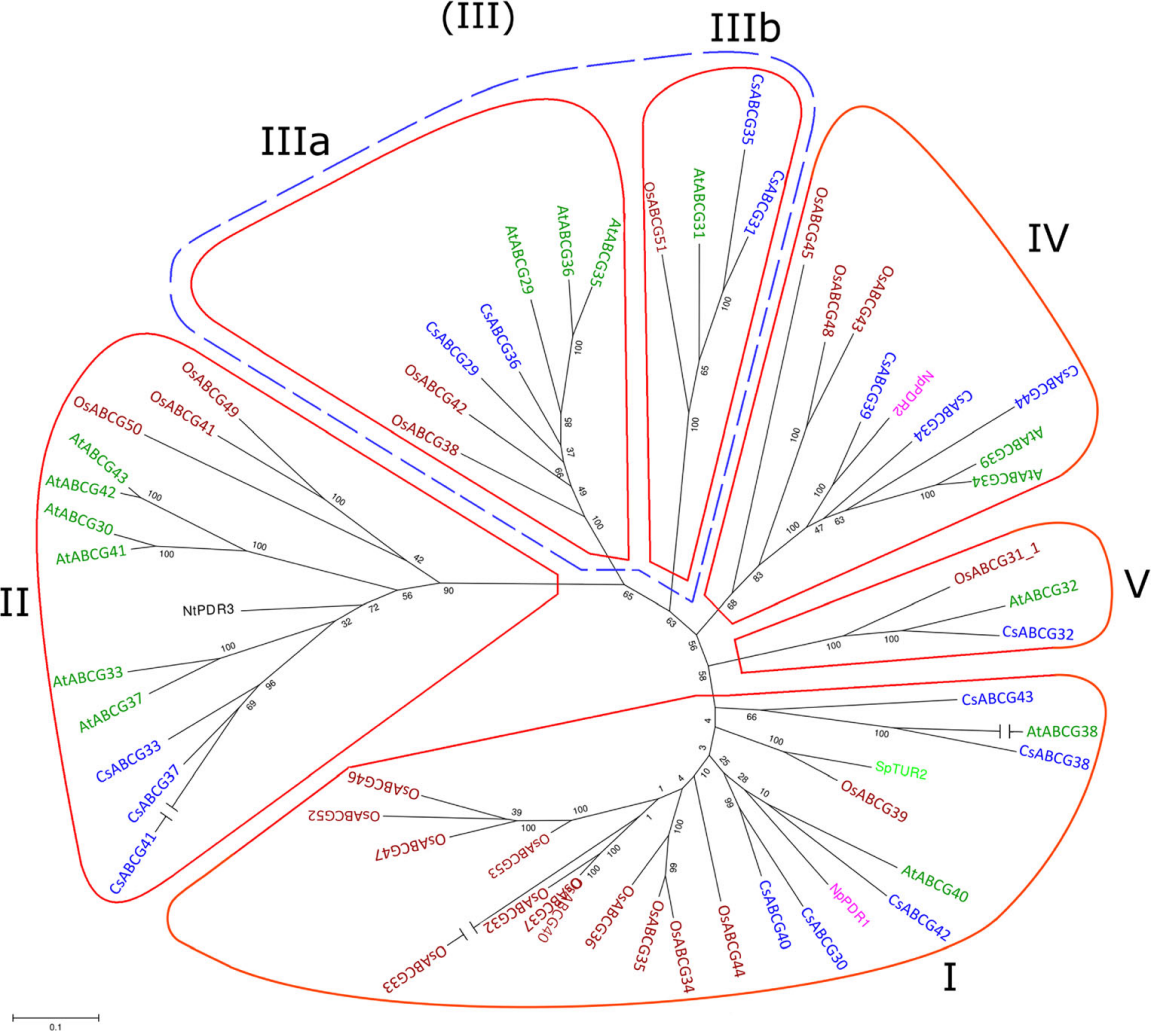
Phylogenetic analysis of the PDR members of ABCG subfamily from plants. Predicted protein sequences were aligned using Clustal W program. The maximum likelihood method (MEGA 5.05 package) was used to construct the unrooted phylogenetic tree with 1000 bootstrap replicates. The lengths of branches are proportional to phylogenetic distances. The accession numbers are as follows: *A. thaliana*, AtABCG29 (DAA00870), AtABCG30 (DAA00869), AtABCG31 (DAA00871), AtABCG32 (DAA00872), AtABCG33 (DAA00873), AtABCG34 (DAA00874), AtABCG35 (DAA00875), AtABCG36 (DAA00876), AtABCG37 (DAA00877), AtABCG38 (DAA00878), AtABCG39 (DAA00879), AtABCG40 (DAA00880), AtABCG41 (DAA00881), AtABCG42 (DAA00882), AtABCG43 (DAA00883); *O. sativa*, OsABCG31 (Q8GU87), OsABCG32 (AAQ01165), OsABCG33 (AJ535214), OsABCG34 (Q7PC80 or DAA00884), OsABCG35 (Q8GU92 or CAD59566 or DAA00885), OsABCG36 (AAQ02685, Q8GU90 or CAD59568), OsABCG37 (Q8GU89 or CAD59569), OsABCG38 (Q7FMW4 or CAD59563), OsABCG39 (Q8U88 or CAD59570), OsABCG40 (DAA00887), OsABCG41 (CAD59575 or Q8GU83), OsABCG42 (Q5Z9S8 or CAD59565), OsABCG43 (Q8GU86), OsABCG44 (Os08g29570), OsABCG45 (CAD59576 or Q8GU82), OsABCG46 (Os09g16290), OsABCG47 (EAZ44308), OsABCG48 (CAD59574 or Q8GU84), OsABCG49 (Os12g13720), OsABCG50 (Os12g32820), OsABCG51 (AAM18755.1), OsABCG52 (B9G300.2), OsABCG53 (EAZ44307.1); *N. plumbaginifolia*, NpPDR2 (AJ831424); *N. tabacum*, NtPDR1 (BAB92011), NtPDR3 (CAH39853); *Spirodela polyrhiza*, SpTUR2 (CAA94437); *C. sativus*, CsABCG36 (GQ374243), CsABCG40 (GQ374244), CsABCG29 (ACHR01006946), CsABCG30 (ACHR01001960), CsABCG31 (ACHR01006356/ACHR01006357), CsABCG32 (ACHR01002610), CsABCG33 (ACHR01000871/ACHR01000872), CsABCG34 (ACHR01006492), CsABCG35 (ACHR01001369), CsABCG37 (ACHR01000873/ACHR01000874), CsABCG38 (ACHR01012132), CsABCG39 (ACHR01008429), CsABCG41 (ACHR01004252), CsABCG42 (ACHR01010133), CsABCG43 (ACHR01000615), CsABCG44 (ACHR01001600).

The sequences of the *PDR* genes have been recently identified (Migocka et al. 2012) in the cucumber genome available in the GenBank under accession number ACHR01000000 (Huang et al. 2009). The putative CsPDR proteins were re-named according to the current nomenclature of the full-size members of the ABCG subfamily of ABC transporters. Hence, the previous CsPDR1-16 proteins are now presented as CsABCG29-44 proteins (Fig. 1). The sequences of full-size ABCG proteins from other plants were retrieved from the TAIR (*A. thaliana*), GenBank (*N. tabacum*, *N. plumbaginifolia*, *Spirodela polyrrhiza*), and Gramene database (*O. sativa*) (Fig. 1). The alignment of all proteins was performed using ClustalW with default parameters and the phylogenetic tree was generated using the MEGA 5.05 software (Tamura et al. 2011) and maximum likelihood method with bootstraps 1000. The putative promoter sequences were extracted from genomic contigs containing *CsABCG* genes based on genes’ start annotations provided by FGENESH analysis. For the detection of *cis* regulatory motifs responsive to hormones, stress, and fungal elicitors, promoter sequences were analyzed using the PlantCARE database. The fragments of 1500 bases upstream of the start codons were considered for analysis.

### DNA Sequencing and Data Analysis

Each PCR amplified gene fragment was purified by agarose gel electrophoresis, ligated into pGEM vector (Promega), and sequenced. The EST sequences were submitted to GenBank and annotated according to the nomenclature given to all *CsABCGs* (*CsPDRs*) previously (Migocka et al. 2012) (Supplementary Table 2). The crossing amplification point (Cp) as well as the relative expression levels (ΔΔCT-method) of cucumber genes were calculated using the LightCycler 480 software 1.5. The significance of differences in transcript copy number was analyzed by Student’s *t* test and ANOVA.

## Results

### Phylogenetic Analysis of Full-Size Cucumber ABCGs

**Fig. 2:**
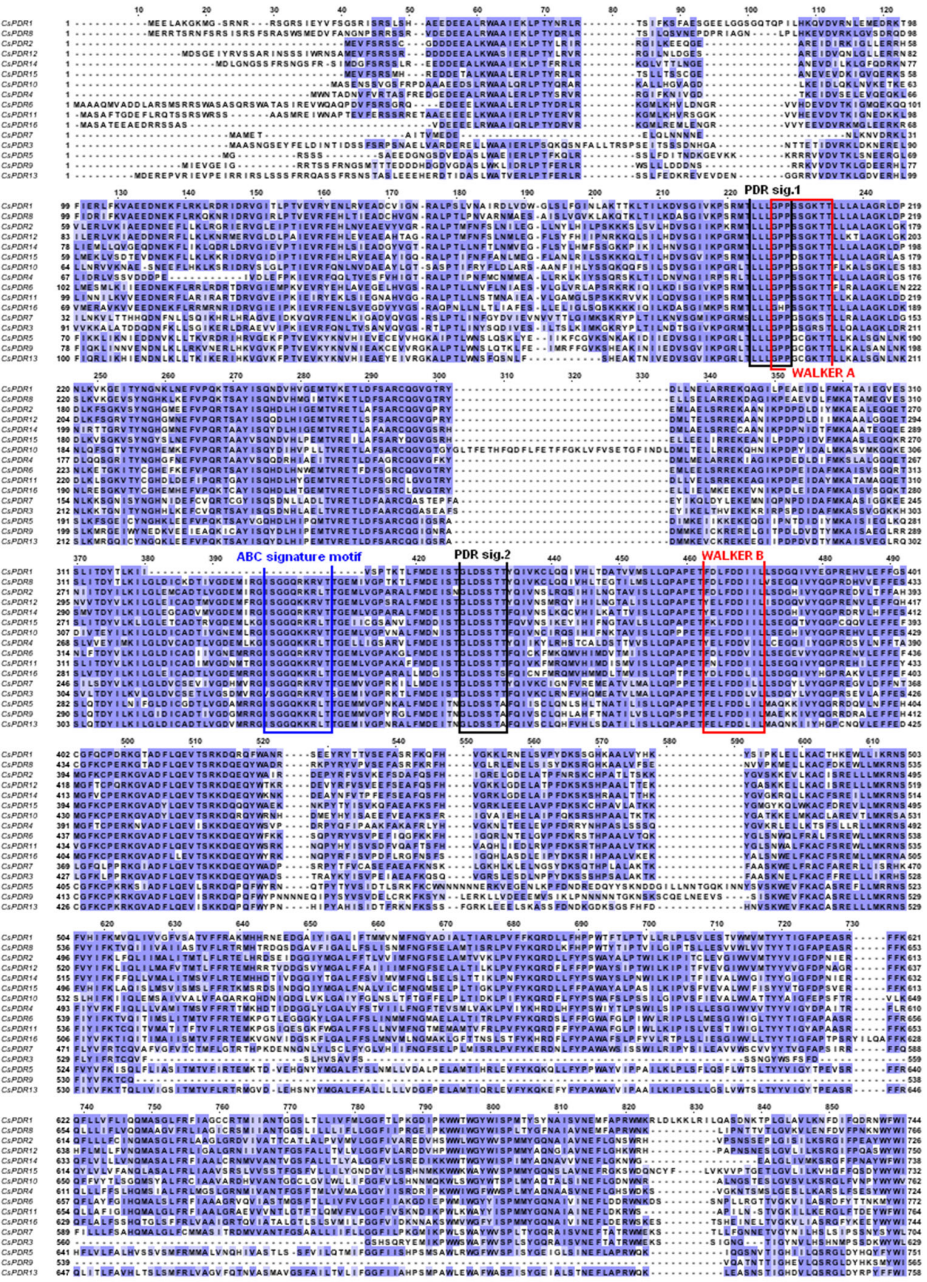

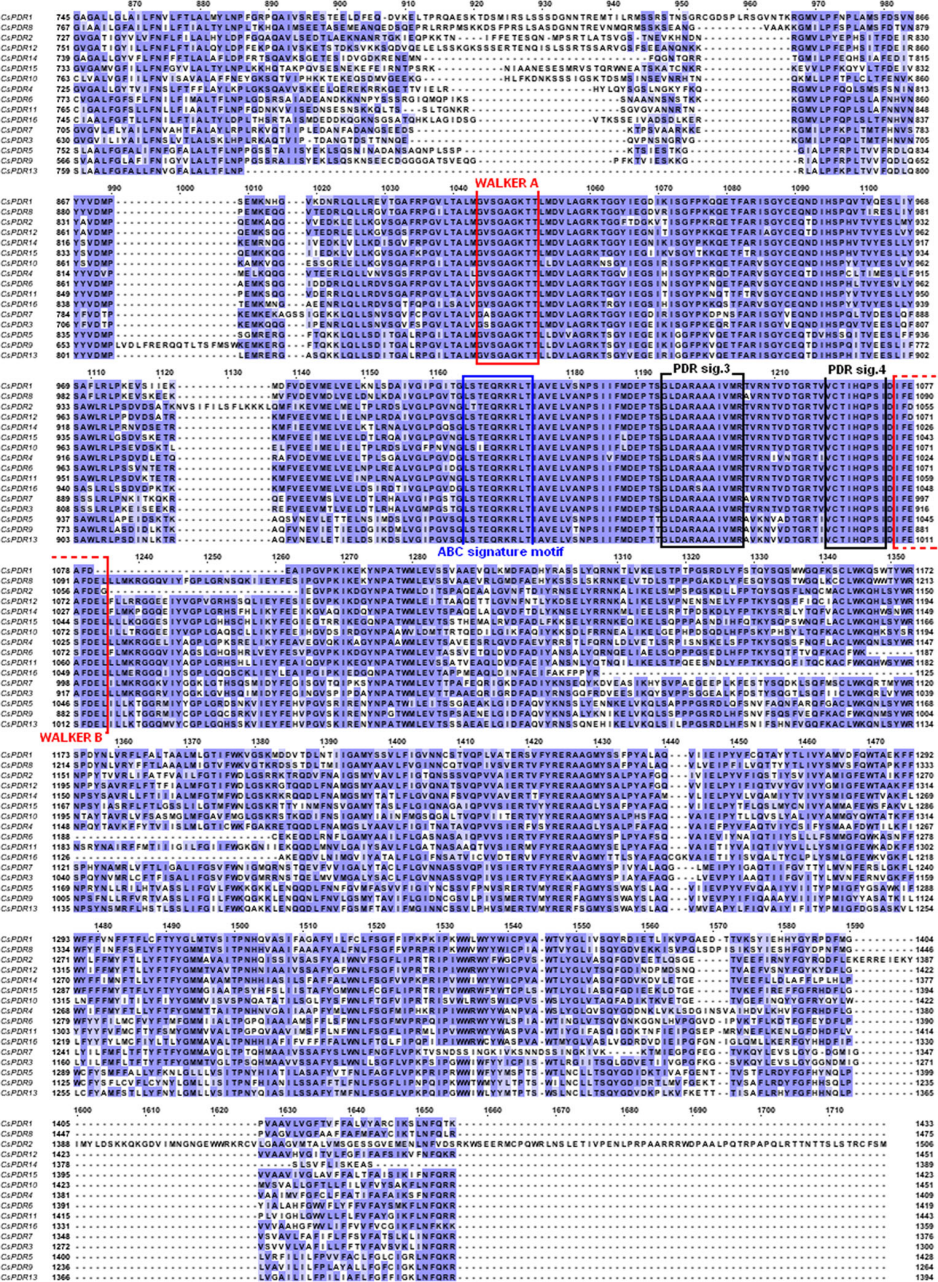
The multiple alignment of all predicted full-size ABCG proteins from cucumber with Clustal W. Sequence stretches representing domains WalkerA, WalkerB, and ABC that are conservative within ABC transporters as well as the characteristic PDR signatures are *boxed*, and the positions exhibiting highly conserved or identical amino acids are *color* shaded

To date, only two genes encoding full-size cucumber ABCG proteins have been cloned to their full length: *CsABCG36* (*CsPDR8*) and *CsABCG40* (*CsPDR12*) (Migocka et al. 2012). The remaining 14 genes encoding full-size ABCG proteins from cucumber have been retrieved from GenBank through the query of the genome of Chinese long cultivar and annotated based on the homology to their orthologs in *A. thaliana* (Migocka et al. 2012). All the putative full-size cucumber ABCG proteins were aligned using ClustalW to determine the residue conservation patterns within the family. This multiple alignment revealed the location of evolutionary conserved domains commonly found in members of the ABC family, such as Walker A and Walker B boxes and two ABC signatures, as well as the four PDR signatures characteristic only for the full-size members of the ABCG subfamily (Fig. 2). Interestingly, no N-terminal ABC signature has been found in putative CsABCG29 protein (Fig. 2). The full-size members of the ABCG subfamily from cucumber and the already annotated homologous proteins from other plants were subjected to phylogenetic analysis using the maximum likelihood method in MEGA software (version 5.05) (Fig. 1). The arrangement of sequences resulting from the analysis clearly shows that the full-size members of ABCG proteins in plants form six separate clusters: I, II, IIIa, IIIb, IV, and V (Fig. 1). This result was inconsistent with previous studies showing the division of the family into five phylogenetic clades (Crouzet et al. 2006; Rea 2007). As shown in Fig. 1, the additional cluster arose from the division of clade III into two separate clades, which have been currently designated as IIIa and IIIb. Cluster I includes most of the *O. sativa* ABCGs (OsABCG32-OsABCG37, OsABCG39, OsABCG40, OsABCG44, OsABCG46, OsABCG47, OsABCG52, and OsABCG53), five cucumber ABCGs (CsABCG30, CsABCG38, CsABCG40, CsABCG42, and CsABCG43), two *A. thaliana* ABCGs (AtABCG38 and AtABCG40), and homologous proteins from *N. plumbaginifolia* (NpPDR2) and *Spirodela polyrrhiza* (SpTUR2). Cluster II contains six *A. thaliana* ABCGs (AtABCG30, AtABCG33, AtABCG37, and AtABCG41-43), three cucumber ABCGs (CsABCG33, CsABCG37, and CsABCG41), three OsABCGs (OsABCG49, OsABCG50), and one homologous protein from *N. tabacum* (NtPDR3). The three *A. thaliana* PDRs, AtABCG29, AtABCG35, and AtABCG36, together with cucumber CsABCG29 and CsABCG36, and rice OsABCG38 and OsABCG42, form cluster IIIa, whereas the four orthologous proteins AtABCG31, OsABCG51, CsABCG31, and CsABCG35 branch into the separate cluster IIIb (Fig. 1). Cluster IV is composed of rice OsABCG43, OsABCG45, and OsABCG48; cucumber CsABCG34, CsABCG39, and CsABCG44; *A. thaliana* AtABCG29 and AtABCG34; and one homologous protein from *N. plumbaginifolia* (NpPDR2). The smallest cluster V is formed by OsABCG31, AtABCG32, and CsABCG32 (Fig. 1).

### Transcriptional Analysis of 14 Cucumber ABCG Genes in Various Cucumber Organs at Two Different Developmental Stages

The function of proteins in plants is significantly determined by their location and regulation upon plant exposure to various conditions. In the first approach to establish these characteristics for cucumber *ABCGs*, the mRNA levels of *CsABCGs* genes were measured in vegetative organs and inflorescences of cucumber plants using RT-PCR. Four organs of young cucumber seedlings and 12 organs of older, flowering plants were used to establish organ expression patterns of all cucumber *CsABCGs*, except for the *CsABCG36* and *CsABCG40* mRNAs, which were analyzed previously (Migocka et al. 2012). Since the ABCG subfamily belongs to the ABC transporter family, which is the largest family of transmembrane proteins in plants and includes members exhibiting a high degree of similarity, the specific primers used for the comparative transcriptional analysis of cucumber PDR members were designed very carefully to avoid cross-hybridizations (Supplementary Table 1).

**Fig. 3:**
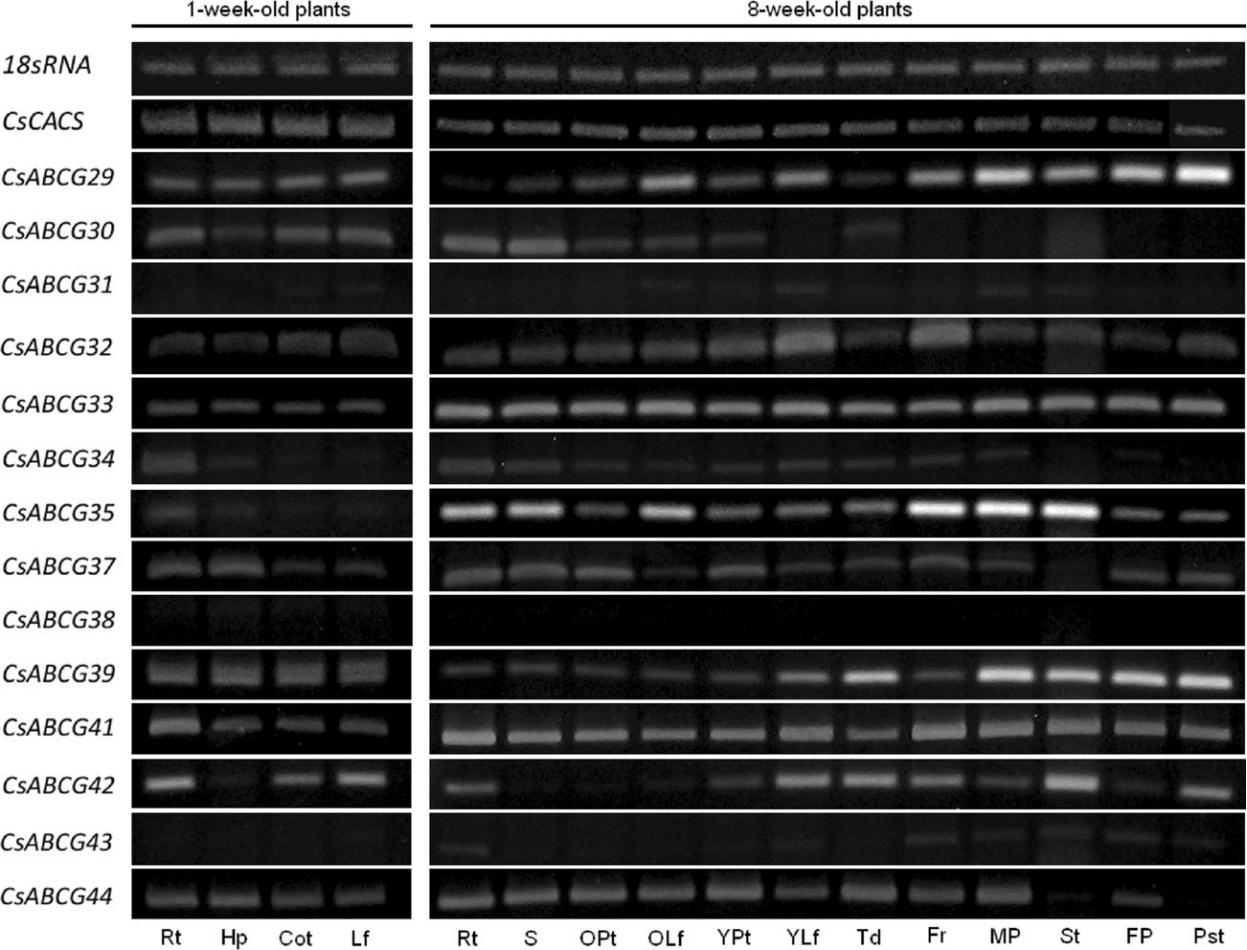
RT-PCR analysis of the organ expression pattern of *CsABCGs* in 1-week-old (young) and 8-week-old (old) cucumber plants. Genes encoding 18s RNA ribosome subunit and clathrin adaptor complex subunit (CACS) were used as internal controls. *Rt* roots, *Hp* hypocotyls, *Cot* cotyledons, *Lf* leafs, *S* stem, *Opt* old petioles, *OLf* old leaves, *YPt* young petioles, *YLf* young leaves, *Td* tendrils, *Fr* fruit, *MP* male perianth, *St* stamen, *FP* female perianth, *Pst* pistil

As shown in Fig. 3, 5 of the 14 *CsABCGs* (*CsABCG32*, *CsABCG33*, *CsABCG37*, *CsABCG39*, and *CsABCG41*) were clearly expressed in all plant organs. Although slightly higher levels of *CsABCG32*, *CsABCG33*, *CsABCG37*, *CsABCG39*, and *CsABCG41* mRNAs were detected in 8-week-old plants than in young seedlings, the distribution in all organs suggests the ubiquitous expression of these cucumber *ABCGs*. In contrast, no expression of *CsABCG38* was detected in both the vegetative organs and inflorescences of cucumbers grown under control conditions (Fig. 3). *CsABCG29* was predominantly expressed in inflorescences and young and old leaves of 8-week-old plants (Fig. 3). In contrast, the transcript level of *CsABCG30* was almost undetectable in inflorescences but highly abundant in all organs of 1-week-old seedlings and in roots and stems of 8-week-old plants (Fig. 3). Contrary to *CsABCG29* and *CsABCG30* transcripts, *CsABCG31* mRNA was only slightly abundant in leaves, male inflorescences, and stamens (Fig. 3). Interestingly, the transcript of the *CsABCG35* gene encoding a protein with high sequence similarity to CsABCG31 was nearly ubiquitously expressed in cucumber (Fig. 3). The level of *CsABCG35* expression was significantly dependent on the stage of plant development, since its mRNA was abundant in organs of the older plants (mostly in fruits and male flowers) but only slightly detected in young cucumber seedlings (Fig. 3). Though the *CsABCG32* transcript was detected in all cucumber organs, it was predominantly expressed in leaves and fruits (Fig. 3). Similar expression patterns were observed for *CsABCG33* and *CsABCG41*. Both genes encoding proteins from the same cluster (Fig. 1) were expressed in each of the studied organs, but their expression levels in older plants were significantly higher when compared to young seedlings (Fig. 3). Though *CsABCG37* encodes a protein belonging to the same phylogenetic cluster, it was predominantly expressed in roots, hypocotyls, and stems of cucumber plants (Fig. 3). *CsABCG34* mRNA was detected in almost all tested organs except for the flowers, and cotyledons and leaves of young cucumber seedlings (Fig. 3). In contrast, the transcript of *CsABCG42* encoding the closest relative of CsABCG34 was abundant in the stamens and pistils of cucumber flowers but was not detected in hypocotyls and stems, or in young and old leaves and their petioles (Fig. 3). Contrary to *CsABCG42*, *CsABCG44* encoding protein from the same cluster was expressed in almost all tested organs except for the stamens and pistils (Fig. 3). Compared to the other expressed cucumber *CsABCGs*, the *CsABCG43* mRNA was present at a very low level only in roots, young leaves, flowers, and fruits of 8-week-old plants (Fig. 3). Altogether, these results clearly indicate that the organ expression patterns of *CsABCG* are not correlated with the cluster assignments of CsABCG proteins.

### Transcriptional Profiling of Cucumber ABCGs in Response to Phytohormones, Plant Growth Regulators, and Sclareolide

**Fig. 4:**
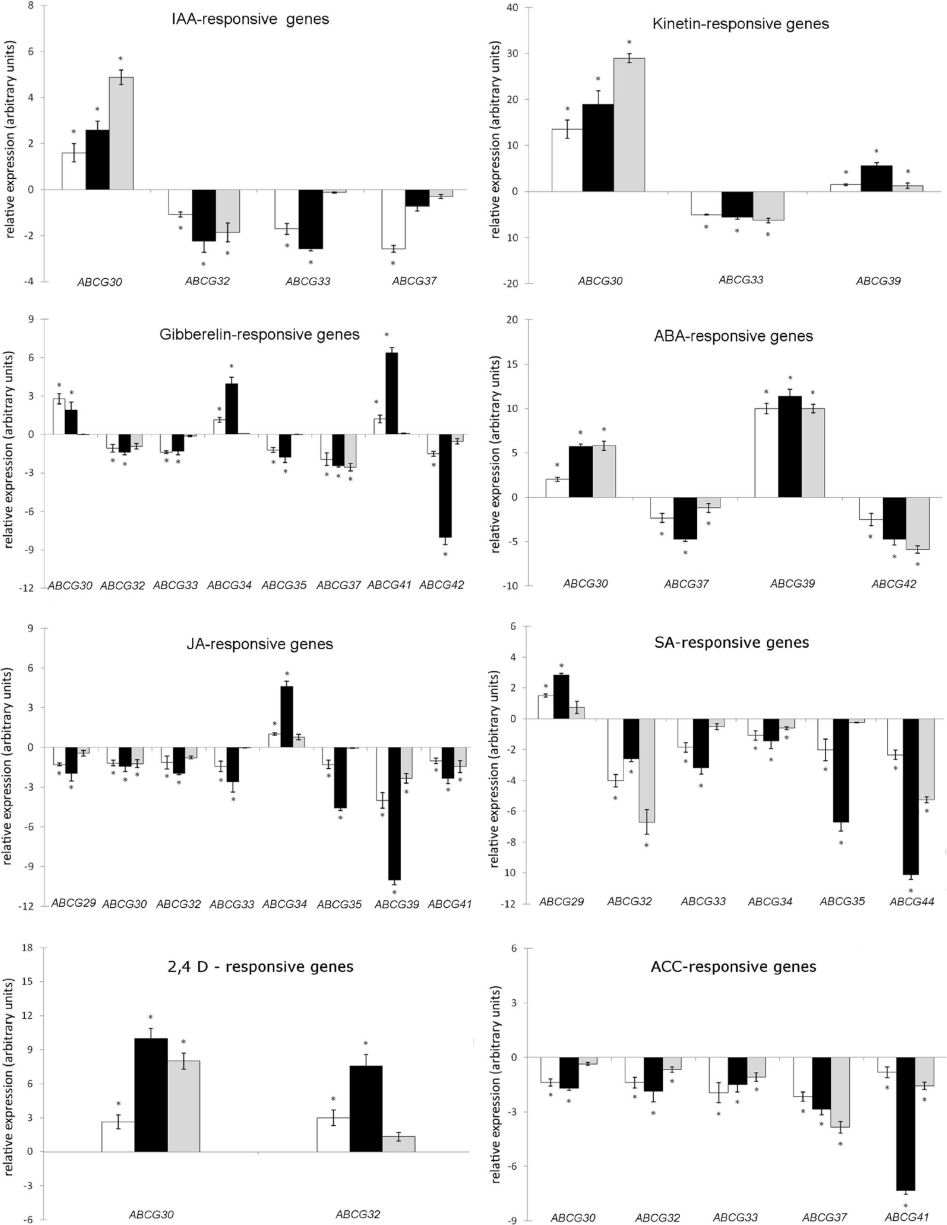
Quantitative real-time PCR analysis *CsABCG* expression under phytohormones and plant growth regulators. Transcripts level was studied in the roots of 1-week-old cucumbers treated with IAA (50 μM), 2,4-D (5 μM), kinetin (50 μM), GA_3_ (50 μM), ABA (50 μM), SA (20 μM), ACC (200 μM), and JA (20 μM) for 4 h (*white bars*), 8 h (*dark bars*) or 12 h (*gray bars*). Plants treated with the equivalent amount of methanol (the solvent of IAA and 2,4-D) or water were used as control. Relative expression levels at control conditions were set to be equal to 0. The values for each treatment are expressed as mean + SD and calculated accordingly. Data are obtained from at least three independent experiments. Significant differences between control and hormone-treated plants are indicated by asterisks (*t* test; *P < 0.05)

**Fig. 5:**
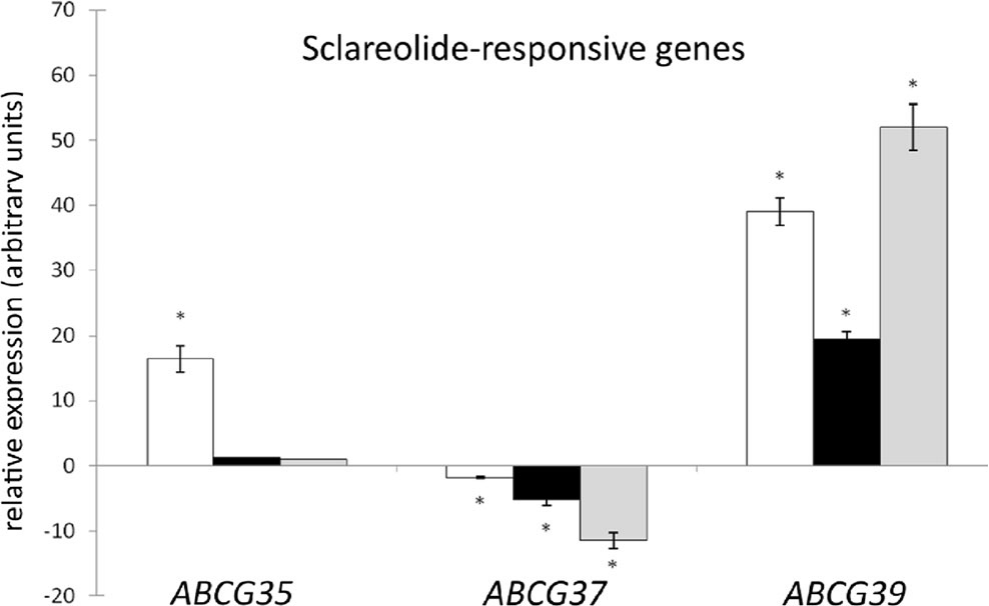
Quantitative real-time PCR analysis of the level of *CsABCG7*, *CsABCG9*, and *CsABCG11* transcripts under sclareolide. The analysis was performed using the roots of 1-week-old cucumbers treated with sclareolide (500 μM) for 4 h (*white bars*), 8 h (*dark bars*), or 12 h (*gray bars*). Plants treated with the equivalent amount of DMSO (the solvent of sclareolide) were used as control. Relative expression levels at control conditions were set to be equal to 0. The values for each treatment are expressed as mean + SD and calculated accordingly. Data are obtained from at least three independent experiments. Significant differences between control and hormone-treated plants are indicated by asterisks (*t* test; *P < 0.05)

The expression of all 14 cucumber *CsABCGs* was further analyzed in cucumber roots grown under phytohormones, plant growth regulators, and sclareolide, a close analog of the antifungal elicitor sclareol, in order to initially dissect the possible relation of root-expressed cucumber *CsABCGs* with phytohormonal signaling or transport or with pathogen resistance. A wide variety of different growth regulators were used in this study, including hormones or hormonal precursors (ACC) involved in the plant response to biotic (SA, JA) and abiotic (ABA, ACC) stresses, as well as the natural and synthetic regulators affecting plant growth and development (IAA, GA, kinetin, 2,4-D). The organ expression assay revealed that 11 cucumber *ABCGs*—*CsABCG29*, *CsABCG30*, *CsABCG32*, *CsABCG33*, *CsABCG34*, *CsABCG35*, *CsABCG37*, *CsABCG39*, *CsABCG41*, *CsABCG42*, and *CsABCG44—*were clearly expressed in the roots of 7-day-old cucumbers grown in control nutrition medium (Fig. 3). Although the transcripts of *CsABCG31*, *CsABCG38*, and *CsABCG43* were not detectable in the same roots, we also included these three genes in further expression analyses to determine whether they are induced by plant growth regulators or sclareolide. Nevertheless, we did not detect any *CsABCG31*, *CsABCG38*, or *CsABCG43* mRNAs in cucumber seedlings during the whole time course (4, 8, and 12 h) of treatment (data not shown). The expression patterns of genes that were significantly affected by different plant growth regulators and sclareolide are presented in Figs. 4 and 5. The response of root-expressed *CsABCGs* to various treatments was different and often dependent on the time course of the experiment. The *CsABCG29* transcript was differentially affected only by two phytohormones: salicylic acid and jasmonic acid (Fig. 4). While SA elevated the *CsABCG29* mRNA level almost 3-fold after 8-h-long treatment, JA reduced gene expression approximately 2-fold in the same time course of the experiment. Contrary to *CsABCG29*, *CsABCG30* expression was differentially affected by nearly every regulator used in the study (Figs. 4). The highest increase in *CsABCG30* mRNA level was observed under 12-h-long kinetin (up to 30-fold) and 2,4-D (up to 10-fold) treatments (Fig. 4). The strongest stimulatory effect of ABA and IAA on *CsABCG30* expression was also observed under the longest treatments of plants with phytohormones (Fig. 4), whereas GA_3_ caused the maximal 3-fold increase in the gene transcript level after the first 4 h of the experiment (Figs. 4). In contrast, JA and ACC down-regulated *CsABCG30* expression approximately 2-fold when compared with the control. Interestingly, the effect of JA and ACC was the highest after the first 8 h of the experiment and was almost completely abolished following the next 4 h of the treatments (Fig. 4). The expression data were in agreement with the analysis of *CsABCG29* and *CsABCG30* promoter sequences. The *cis*-acting regulatory elements involved in salicylic acid and jasmonate responsiveness were identified within the promoter of *CsABCG29*, whereas the *CsABCG30* promoter contained two regulatory elements responsive to jasmonate (CGTCA and TGACG motifs) as well as the motifs responsive to gibberellins and auxins (Table 1). Similarly to *CsABCG30*, *CsABCG32* expression was also affected by several plant growth regulators. The significant increase in *CsABCG32* mRNA (8-fold) was observed only upon 4- and 8-h-long exposure of plants to the auxinic herbicide 2,4-D, whereas phytohormones and the ethylene precursor decreased gene expression over 2-fold (JA, GA_3_, IAA, ACC) or even up to 7–8-fold (SA) in different time courses of experiments (Fig. 4). The inhibitory effect of ACC, JA, and GA_3_ on *CsABCG32* mRNA level was the most pronounced during the 4 and 8 h of treatments, whereas IAA- and SA-dependent down-regulation of the gene expression was the greatest in the 8- and 12-h-long experiments (Fig. 4). Nevertheless, the hormone-mediated signals clearly acted as repressors of *CsABCG32* transcription. Consistent with the expression profile, multiple elements involved in gibberellin, auxin, jasmonate, or salicylate responsiveness have been identified within the *CsABCG32* promoter (Table 1), confirming that this regulatory region of the cucumber gene may be the end point of the phytohormone-induced signaling pathway for *CsABCG32* repression in cucumber roots. Similarly to *CsABCG32*, the expression of *CsABCG33* was markedly down-regulated by most of the growth regulators used in the study. The decrease in *CsABCG33* mRNA was the most apparent under kinetin (up to 5-fold decrease) and salicylic acid (3-fold decrease) treatments (Fig. 4). However, the effect of kinetin was not dependent on the time course of experiments, whereas the effect of SA was significant only during the first 8 h of treatment (Fig. 4). In addition, ACC, GA_3_, and JA also down-regulated *CsABCG33* expression (up to 2-fold) (Fig. 4). Similarly to SA, the inhibitory effect of ACC, GA_3_, and JA was the most pronounced after the first 8 h of plant exposure to regulatory compounds. The observed changes in *CsABCG33* expression were consistent with the presence of regulatory elements responsive to ethylene, gibberellins, and jasmonate in the *CsABCG33* promoter (Table 1). The *CsABCG34* transcript was also slightly (2-fold) reduced by SA, but the greatest change in gene expression was observed under JA and GA_3_ (4-fold increase) (Fig. 4). The phytohormones had the most pronounced effect on *CsABCG34* expression after the first 8 h of treatments (Fig. 4). Interestingly, the regulatory region of *CsABCG34* contains only the regulatory elements responsive to gibberellins, TC-rich repeats involved in the stress response, and the MYB binding site involved in drought inducibility (Table 1), suggesting that both JA and SA may affect cucumber gene expression via an additional indirect regulatory mechanism. Similarly to *CsABCG34*, expression of *CsABCG35* was significantly affected by JA, SA, and GA_3_. All three compounds caused a significant 2-fold (GA), 4-fold (JA), or 6-fold (SA) decrease in *CsABCG35* transcript level after the first 8 h of treatments (Figs. 4 and 5). In contrast, sclareolide increased *CsABCG34* expression up to 4-fold in the first 4 h of the experiment (Fig. 5). The stimulatory effect of diterpene was markedly abolished with time. Consistent with the expression assay, sequence analysis revealed the presence of two regulatory elements responsive to jasmonate as well as elements responsive to salicylate, gibberellin, and a fungal elicitor within the *CsABCG35* promoter (Table 1). It has previously been shown that JA, SA, and GA_3_ levels in plants are significantly changed during mycorrhizae formation, indicating a function of these phytohormones in plant-fungi interaction (Barea and Azcon-Aguilar 1982; Stumpe et al. 2005; Yang et al. 2005). The results from expression and promoter analysis suggest that *CsABCG35* may be involved in the plant response to fungi and reveal the complex phytohormone-mediated and fungal elicitor-mediated regulatory mechanism of transcription of genes involved in plant-fungi interaction. Contrary to *CsABCG35*, *CsABCG37* was markedly (up to 11-fold) down-regulated upon sclareolide treatment, and the effect of diterpene increased with the time of the experiment (Fig. 5). In addition to sclareolide, the phytohormones GA_3_, ABA, and IAA as well as the ethylene precursor ACC also decreased *CsABCG37* expression up to 2.5-, 3.7-, 4-, and 3.8-fold, respectively (Fig. 4). However, the effect of GA_3_ and ACC increased in time, whereas the effect of ABA and IAA was the most pronounced after 8 h of treatment (Fig. 4). The alterations in *CsABCG37* expression could be explained by the presence of multiple regulatory elements responsive to ABA, gibberellins, ethylene, and fungal elicitor in the gene promoter (Table 1). It has been well described that ethylene and ABA are involved in plant susceptibility to the pathogenic fungus *Botrytis cinerea* (Sharon et al. 2007). The results from expression assay and promoter analysis suggest that complex regulation of the cucumber response to fungal attack may include CsABCG37, a fungal elicitor, and phytohormones such as ABA, GA_3_, and ethylene. Though IAA also significantly affected *CsABCG37* transcription, the auxin-responsive regulatory elements were absent in the promoter sequence of the cucumber gene (Table 1), suggesting the influence of auxin on *CsABCG37* transcription via a more complex and indirect mechanism.

**Table 1:** Hormone-end stress-responsive *cis*-regulatory elements identified within the upstream regions of cucumber genes encoding full-size ABCG proteins. The conserved *cis*-regulatory elements were found in the 1500-nt (nucleotide) sequence upstream of each the genes subjected to analysis. The *cis*-elements and the related functional descriptions were based on the prediction results by PlantCARE (http://bioinformatics.psb.ugent.be/webtools/plantcare/html/) (Lescot et al. 2002)

*Cis*-element	Description	Cucumber genes encoding root-expressed *CsABCGs*
ABRE	*Cis*-acting element involved in the abscisic acid responsiveness	*CsABCG37*, *CsABCG38*, *CsABCG39*, *CsABCG42*
MBS	MYB binding site involved in drought-inducibility	*CsABCG32*, *CsABCG33*, *CsABCG34*, *CsABCG35*, *CsABCG37*, *CsABCG39*, *CsABCG43*, *CsABCG44*
Box-W1	Fungal elicitor responsive element	*CsABCG41*, *CsABCG35*, *CsABCG37*, *CsABCG39*
CGTCA-motif	*Cis*-acting regulatory element involved in the MeJA-responsiveness	*CsABCG41*, *CsABCG30*, *CsABCG32*, *CsABCG35*, *CsABCG36*
TCA-element	*Cis*-acting regulatory element involved in salicylic acid responsiveness	*CsABCG43*, *CsABCG29*, *CsABCG35*, *CsABCG44*
CRM	Cytokinin response motif	
P-box	Giberellin-responsive element	*CsABCG35*, *CsABCG41*, *CsABCG43*, *CsABCG44*
GARE-motif	Giberellin-responsive element	*CsABCG32*, *CsABCG33*, *CsABCG30*, *CsABCG34*, *CsABCG37*, *CsABCG39*, *CsABCG42*, *CsABCG43*, *CsABCG44*
TC-rich repeats	*Cis*-acting element involved in defense and stress responsiveness	*CsABCG30*, *CsABCG29*, *CsABCG32*, *CsABCG33*, *CsABCG34*, *CsABCG35*, *CsABCG37*, *CsABCG38*, *CsABCG39*, *CsABCG43*, *CsABCG44*
TGACG-motif	*Cis*-acting regulatory element involved in the MeJA-responsiveness	*CsABCG41*, *CsABCG30*, *CsABCG29*, *CsABCG32*, *CsABCG33*, *CsABCG35*, *CsABCG39*
AuxRR-core	*Cis*-acting regulatory element involved in auxin responsiveness	*CsABCG30*, *CsABCG32*
ERE	ethylene-responsive element	*CsABCG41*, *CsABCG33*, *CsABCG37*

Similarly to *CsABCG35* and *CsABCG37*, the expression of *CsABCG39* was also affected by the analog of sclareol. Compared to *CsABCG35* and *CsABCG37*, sclareolide increased *CsABCG39* transcription to a considerably greater extent, causing a 40-fold and 55-fold increase in the mRNA level after 4 and 12 h of treatment, respectively (Fig. 5). In addition, the transcript of this gene was also markedly elevated by ABA (over 11-fold), 2,4-D (over 5-fold), and kinetin (over 5-fold), whereas JA greatly (over 13-fold) down-regulated *CsABCG39* (Fig. 4). Similarly to *CsABCG37*, regulatory elements responsive to ABA, jasmonate, and fungal elicitor were found in the *CsABCG39* promoter sequence (Table 1), suggesting similar function of both cucumber ABCG37 and ABCG39 proteins in plant-fungi interaction including ABA and JA signaling. Interestingly, the regulatory motifs responsive to cytokinins were absent in the *CsABCG39* promoter (Table 1). Both 2,4-D and kinetin are known to strongly promote plant cell divisions, which can be very intensive during colonization of plant roots by fungi. Hence, 2,4-D and kinetin may affect *CsABCG39* expression in an additional indirect mechanism, which could be a part of the complex response of plants to fungi.

*CsABCG41* was transcriptionally down-regulated by JA (over 3-fold) and ACC (over 7-fold) and up-regulated by GA_3_ (over 6-fold) (Fig. 4), consistent with the presence of regulatory motifs involved in responsiveness to jasmonate (two elements), gibberellins, and ethylene in its promoter sequence (Table 1). The effect of the three regulators was the most pronounced after 8 h of the experiment. *CsABCG42* was significantly down-regulated by ABA (9-fold) and GA_3_ (almost 5-fold) (Fig. 4). The effect of ABA increased with time, whereas GA_3_ was the most effective after 8 h of treatment. *Cis*-acting regulatory elements for GA_3_ (GARE motif) and ABA (ABRE-motif) were present in the *CsABCG42* promoter (Table 1), confirming the function of this gene in gibberellin and ABA-mediated responses. The root expression of *CsABCG44* was affected only by SA supply, which caused over a 10-fold decrease in the gene transcript after 8 h of plant treatment (Fig. 4). Interestingly, the analysis of the *CsABCG44* promoter revealed the presence of regulatory elements responsive to SA but also motifs involved in gibberellin perception (Table 1). The regulation of gene transcription not only is dependent on the promoter structure but also includes a wide range of mechanisms, such as chromatin re-modeling, which can be induced by histone modification or DNA methylation. It is also dependent on the type of tissue or organ used for the analysis. *CsABCG44* was expressed in almost every cucumber organ except the stamens and pistils (Fig. 3). Hence, we may suspect that either the gibberellin-responsive elements were not available for binding by trans-regulatory elements induced by GA_3_ or that the GA_3_-mediated regulation of *CsABCG44* transcription occurs in different cucumber organs.

In summary, the analysis revealed quite different expression patterns of root-expressed *CsABCGs* in response to plant growth regulators and antifungal diterpene. In addition to phytohormone and elicitor responsive regulatory elements, we found TC-rich repeats involved in defense and stress responsiveness in 12 of the 14 root-expressed *CsABCGs* (Table 1). Therefore, the function of the PDR family in cucumber may indeed be related to the stress response mediated by phytohormonal signals or antimicrobial elicitors. Based on their transcriptional profile, the analyzed genes might be grouped into IAA-responsive (*CsABCG30*, *CsABCG32*, *CsABCG33*, *CsABCG37*), kinetin-responsive (*CsABCG30*, *CsABCG33*, *CsABCG39*), gibberellin-responsive (*CsABCG30*, *CsABCG32*, *CsABCG33*, *CsABCG34*, *CsABCG35*, *CsABCG37*, *CsABCG41*, *CsABCG42*), ABA-responsive (*CsABCG30*, *CsABCG37*, *CsABCG39*, *CsABCG42*), jasmonate-responsive (*CsABCG29*, *CsABCG30*, *CsABCG32*, *CsABCG33*, *CsABCG34*, *CsABCG35*, *CsABCG37*, *CsABCG39*, *CsABCG41*), salicylic acid-responsive (*CsABCG29*, *CsABCG32*, *CsABCG33*, *CsABCG34*, *CsABCG35*, *CsABCG44*), 2,4-D-responsive (*CsABCG30*, *CsABCG32*), ACC(ethylene)-responsive (*CsABCG30*, *CsABCG32*, *CsABCG33*, *CsABCG37*, *CsABCG41*), and sclareolide-responsive (*CsABCG35*, *CsABCG37*, *CsABCG39*) genes (Figs. 4 and 5).

## Discussion

The *ABCG* genes identified in the cucumber genome encode full-size ABC proteins with typical reverse configuration of NBD-TMD domains (NBD-TMD)_2_. Using available sequences from different plant species, it has been previously shown that the full-size ABCGs from plants form five separate phylogenetic clusters (I–V) with cluster I containing the so far studied SpTUR2, AtABCG40/AtPDR12, NpPDR1 and most rice ABCGs (Crouzet et al. 2006; Moons 2008). The novel phylogenetic tree constructed based on the predicted protein sequences from tobacco, rice, *Arabidopsis*, *Spirodela*, and cucumber clearly shows that the former cluster III can be further divided into two separate clusters and finally provides six different phylogenetic subgroups of full-size ABCGs from plants (Fig. 1). Most rice ABCGs (11) are grouped within cluster I, whereas *A. thaliana* homologs are predominantly (6) located in cluster II. Similarly to rice ABCGs, the largest number of cucumber ABCGs (5) are present in cluster I, whereas the others are quite equally distributed between the remaining clusters III, IV, and V (1–3 sequences in each cluster). This clustering confirms the previous indication that the divergence between plant full-size ABCGs occurred before the separation between monocots and dicots and that the patterns of gene duplication in different plant ancestors were different (Crouzet et al. 2006). When compared to OsABCGs, there is no cluster where the multiplication of cucumber or *A. thaliana* homologs was higher than five and six, respectively, which may suggest that the range-wide differentiation of dicot ABCGs was accompanied by stronger differentiation of their physiological functions and/or the ability to transport additional substrates. Nevertheless, further studies of the full-size plant ABCGs are required to evaluate this hypothesis.

The vast majority of plant *ABCG* genes characterized to date are associated with the plant response to stress conditions and phytohormone transport and/or signaling (Crouzet et al. 2006; Eichhorn et al. 2006; Kang et al. 2010; Moons 2008; Ruzicka et al. 2010; Smart and Fleming 1996; Strader and Bartel 2009). Moreover, the expression of the two recently analyzed cucumber *ABCGs*, *CsABCG36* (*CsPDR8*) and *CsABCG40* (*CsPDR12*), was strongly affected by some plant growth regulators related to stress response, suggesting the contribution of members of the cucumber *ABCG* subfamily to the hormone-mediated response to environmental constraints (Migocka et al. 2012). In order to gain a better view of the expression pattern of root-expressed *ABCGs*, in this work we studied the effect of plant growth regulators and an analog of an antifungal elicitor on the distribution of transcripts of the remaining 14 cucumber *ABCGs*. The obtained results revealed a significantly different regulation pattern shown by hormonal compounds and sclareolide among *CsABCGs* belonging to the same clusters, suggesting functional diversity of the cucumber paralogs.

### CsABCGs from Cluster I

Of the five *CsABCG* genes encoding proteins from cluster I, *CsABCG38* and *CsABCG43* were not expressed in roots, and *CsABCG40* was studied earlier; hence, only the results from *CsABCG30* and *CsABCG42* expression are presented. Kinetin considerably enhanced *CsABCG30* transcript accumulation; however, the stimulatory effect of IAA, 2,4-D and GA_3_ on gene expression was also significant. Though all three compounds belong to quite different groups of hormones, they seem to synergistically coordinate some metabolic pathways. Kinetin represents cytokinins, a class of hormones which are known to promote intense cellular divisions; auxins are essential for proper coordination of plant growth and development, whereas gibberellins are known to stimulate cell elongation. *CsABCG40*, the closest relative of *CsABCG30*, was also up-regulated by kinetin, although to a lesser extent (Migocka et al. 2012). Contrary to *CsABCG30*, the *CsABCG40* transcript level was not affected by IAA and GA3, but it was considerably elevated by another compound promoting cell division, the herbicide 2,4-D (Migocka et al. 2012). The results suggest that both genes might be involved in the processes associated with plant growth and development. It is not known, however, whether they contribute to direct transport of hormonal compounds or respond to hormonal signaling indirectly, through other mechanisms. In addition, *CsABCG30* and *CsABCG40* expression was similarly up-regulated by ABA, the hormone acting as a mediator in controlling adaptive plant responses to environmental stresses, such as drought, chilling, salinity, or pathogen infection. It has been recently shown that the homologous protein AtABCG40 from *A. thaliana* mediates the uptake of ABA into the cell through the active transport of this hormone across the plasma membrane and thus contributes to ABA-dependent plant reactions to stress conditions (Kang et al. 2010). In addition, the third cucumber ABCG belonging to the same cluster, CsABCG42, was also significantly affected by ABA. ABA is a carotenoid-derived sesquiterpene. Another terpenoid, the antimicrobial sclareol, was found to be a substrate transported by the NpABC1 from *N. plumbaginifolia* and SpTUR2 from *Spirodela polyrrhiza* (Jasinski et al. 2001; van den Brule et al. 2002), both belonging to cluster I of the full-size ABCGs. Hence, it is possible that the members of cluster I contribute to the transport of some terpenoids, including ABA and sclareol. The transcripts of two genes encoding proteins from cluster I—rice *OsABCG33* and cucumber *CsABCG38*—were not detected in any of the tested organs, indicating that both genes are either pseudogenes or are expressed under yet unidentified endogenous or exogenous factors (Moons 2008). For instance, the closest *CsABCG38* homolog from *A. thaliana*, *AtABCG38*, is expressed only in siliques (van den Brule and Smart 2002). The expression of the last cucumber protein from cluster I, *CsABCG43*, was clearly developmentally regulated, since the transcript of this gene was detected only in 8-week-old plants (Fig. 3), and was not induced in the roots of young seedlings under plant growth regulators and sclareolide (data not shown). The different organ expression pattern of cucumber genes encoding ABCGs belonging to this cluster suggests that they are not redundant. Indeed, the differences in expression profile under plant growth regulators indicate that either different regulatory mechanisms or functional diversification characterizes the ABCGs of cluster I.

### CsABCGs from Cluster II

Three cucumber full-size ABCG proteins—CsABCG33, CsABCG37, and CsABCG41—have been classified in cluster II. However, genes encoding these proteins showed different expression patterns under plant growth regulators and sclareolide. *CsABCG33* and *CsABCG37* were repressed by multiple phytohormones. In addition, *CsABCG37* was down-regulated by sclareolide. In comparison, *CsABCG41* was antagonistically regulated by the regulatory compounds*.* However, the common feature of cucumber *ABCGs* from cluster II was the significant alteration of their expression by GA_3_ and the ethylene precursor ACC, suggesting their function in signaling pathways mediated by gibberellins and ethylene. However, the different response of these genes to other phytohormones and sclareolide suggests functional diversification or a differential mode of regulation of *ABCGs* from cluster II. Beside cucumber *ABCGs*, a few genes encoding proteins of this cluster have already been studied. *A. thaliana AtABCG37 (AtPDR9)* encodes the transporter for structurally and functionally diverse compounds including IAA precursors (Ruzicka et al. 2010). *AtABCG37* expression was not affected by IAA, but it was up-regulated by auxin precursors (Ruzicka et al. 2010). Similarly to *CsABCG33* and *CsABCG37*, rice *OsABCG41* encoding a protein from cluster II was up-regulated by IAA (Moons 2008). Hence, members of cluster II can be involved in the transmembrane transport of auxin or auxin-related compounds. In addition to cucumber *CsABCG33* and *CsABCG41*, the genes encoding proteins from this cluster (OsABCG41, NtPDR3) were also affected by salicylic and jasmonic acids (Ducos et al. 2005; Moons 2008), suggesting their physiological function in the SA-dependent or JA-mediated plant response to environmental stimuli. Jasmonates proved to be important in the wounding response and secondary metabolite synthesis, whereas salicylic acid regulates the local apoptotic hypersensitivity response (HR) and systemic acquired resistance (SAR) under biotic stress. Both hormones are highly important for the defensive armory of plants. The response of the members of cluster II to SA and to an antifungal elicitor analog (*CsABCG37*) indicates the possible role of these proteins in plant-pathogen interactions. Additionally, the cucumber genes encoding CsABCG33, CsABCG37, and CsABCG41 were clearly expressed in all cucumber organs, suggesting that they encode proteins with essential functions for plant cells. The obtained results suggest that members of cluster II may represent a constitutive defense pathway in cucumber plants.

### CsABCGs from Cluster IIIa

Clade IIIa is differentiated from the division of the former cluster III and includes two cucumber ABCG proteins, CsABCG29 and CsABCG36 (Fig. 1). *CsABCG36* expression was analyzed previously (Migocka et al. 2012), so in this work only the distribution of the *CsABCG29* transcript was studied. *CsABCG29* mRNA was the most abundant in old leaves, flowers, and fruits of cucumber plants. However, the SA treatment significantly enhanced *CsABCG29* expression also in the roots of young seedlings. Similarly to ethylene, salicylic acid influences expression of genes associated with senescence and fruit yield (Vlot et al. 2009). Hence, the highest accumulation of the *CsABCG29* transcript in organs associated with SA synthesis and action correlated with the response of the gene to SA treatment, indicating the involvement of CsABCG29 in SA-mediated pathways such as plant organs’ maturation and senescence. *OsABCG42* (*OsPDR12*), a closely related gene from rice, was also strongly expressed in old leaves, and up-regulated by JA, cytokinins, and redox perturbations which accompany the aging process (Moons 2008). Contrary to SA, JA negatively affected *CsABCG29* expression. The antagonistic regulation of genes by SA and JA has already been demonstrated for genes encoding PR (pathogenesis-related) proteins (Niki et al. 1998) and rice ABC proteins (Moons 2008) and confirms the opposing influence of both hormonal compounds on some metabolic pathways. *CsABCG36* encoding protein from the same cluster was also up-regulated by salicylic acid. However, contrary to *CsABCG29*, *CsABCG36* exhibited quite a different organ expression profile and was additionally induced by ACC (Migocka et al. 2012), indicating that *CsABCG29* and *CsABCG36* are not functionally redundant. Moreover, the *CsABCG36* transcript was additionally altered under ABA treatment, indicating a more complex function for CsABCG36 protein in hormone-mediated plant reactions (Migocka et al. 2012).

### CsABCGs from Cluster IIIb

Two of the four proteins belonging to cluster IIIb are cucumber CsABCG31 and CsABCG35 (Fig. 1). A very low level of *CsABCG31* transcript was observed only in cotyledons, leaves, and modified leaves (perianth, stamen) of cucumber plants (Fig. 3). The gene transcript was not detected in roots of young seedlings grown under control conditions (Fig. 3) or supplied with growth regulators or sclareolide (data not shown). The closely related *A. thaliana AtABCG31* was also expressed only in leaves (specifically in stomata cells) and leaf derivatives (Galbiati et al. 2008; van den Brule and Smart 2002) and was shown to be involved in ABA-dependent signal transduction within stomata (Galbiati et al. 2008). In contrast, the transcript of the related cucumber gene *CsABCG35* was detected in roots and was markedly affected by phytohormones GA_3_, SA, and JA as well as by sclareolide, compounds involved in signaling pathways providing resistance to herbivorous insects and pathogen or plant-mycorrhizal fungi interaction (Barea and Azcon-Aguilar 1982; Stumpe et al. 2005; Thaler et al. 2002; Yang et al. 2005). Interestingly, a closely related gene from rice (*OsABCG33/OsPDR14*) was not affected by plant growth regulators (Moons 2008). Such a different organ expression profile as well as the variable response to hormonal and elicitor-mediated signals indicates differentiation of the function and regulatory mechanisms of the ABCG proteins from cluster IIIb.

### CsABCGs from Cluster IV

Cluster IV includes three cucumber ABCG proteins: CsABCG34, CsABCG39, and CsABCG44 (Fig. 1). The genes encoding all three proteins displayed quite different patterns of expression under regulatory compounds. While *CsABCG44* expression was affected only by SA, the transcripts of *CsABCG34* and *CsABCG39* were markedly altered by SA, JA, and GA. In addition, *CsABCG39* expression was greatly up-regulated by sclareolide, indicating its role in plant-fungi interaction. Salicylic acid and jasmonate antagonistically influenced *CsABCG34* and *CsABCG35* expression, suggesting a different role of both cucumber genes in SA- and JA-mediated pathways in plant cells. The opposing influence of regulators was also characteristic for *CsABCG39*. The expression of rice *ABCG* genes encoding proteins from the same cluster was also significantly altered by salicylic and jasmonic acids (Moons 2008). In general, genes encoding proteins from cluster IV were predominantly affected by stress-related compounds, suggesting the involvement of members from this cluster in different stress-induced responses. Contrary to cucumber *CsABCGs*, the expression of a gene encoding a closely related protein, NtPDR2 from *N. plumbaginifolia*, was not affected by any of the hormones typically involved in biotic and abiotic stress responses (Trombik et al. 2008). Moreover, unlike NpPDR1, the NpPDR2 expression was not altered upon *B. cinerea* infection (Trombik et al. 2008). Hence, cluster IV contains the constitutive, inducible, and repressible full-size ABCG proteins. Moreover, it has been recently shown that AtABCG39, the closest homolog of CsABCG44 in *A. thaliana*, contributes to xenobiotic (paraquat) import into plant cells (Xi et al. 2012). Thus, the function and regulation of ABCG proteins from cluster IV appear to be complex and very diverse.

### CsABCGs from Cluster V

The smallest cluster V contains only one cucumber PDR protein, CsABCG32 (Fig. 1). *CsABCG32* was clearly expressed in all cucumber organs (Fig. 3), indicating a putative function of CsABCG32 in basic metabolic processes of plant cells. However, the transcript of this gene was significantly decreased under both the hormones involved in growth in development and the stress-related regulatory compounds. Therefore, CsABCG32 can also be involved in multiple plant responses to environmental stimuli. *OsABCG31* (*OsPDR6*), encoding a close homolog of CsABCG32, was also up-regulated by stress-related phytohormone ABA (Moons 2008), suggesting the involvement of plant ABCGs from cluster V in the plant reaction to stress conditions.

## Conclusions

In this article, we present the structural and phylogenetic analysis of the full-size members of the ABCG subfamily in cucumber and propose a novel improved phylogeny for the full-size ABCGs from plants. In addition, we show that some *CsABCGs* are constitutively expressed throughout the cucumber plant and development, whereas others are organ-specific or developmentally regulated. Real-time expression analysis revealed that the root-expressed *CsABCGs* are differentially regulated by plant growth regulators and sclareolide and suggests that phytohormone- and sclareolide-mediated regulatory mechanisms for the ABCGs are not cluster-specific. Overall, the analysis indicates functional and/or regulatory diversification of the full-size ABCGs from cucumber. The data from this work will be helpful in further characterization of the *ABCG* genes in cucumber and in explaining their regulatory mechanisms.

## Electronic supplementary material

Below is the link to the electronic supplementary material.

ESM 1 (DOC 40 kb)

ESM 2 (DOC 47 kb)
