# Supplementary material for: Genes Encoding Cucumber Full-Size ABCG Proteins Show Different Responses to Plant Growth Regulators and Sclareolide
Source: Plant Mol Biol Report. 2015 Nov 14;34:720–36. doi: 10.1007/s11105-015-0956-9 (PMC4923091; doi:10.1007/s11105-015-0956-9)
Supplement: Supplementary file 1 — (DOC 40 kb) [file 11105_2015_956_MOESM1_ESM.doc]

**Supplementary Table 1**

**The list of primers used for *CsABCG*** expression analyzes.

| Gene *CsABCG* | | Forward primer  (5’-3’) | Position in gene | Reverse primer  (5’-3’) | Position in gene | Amplicon size (bp) | Tm  [°C] |
| --- | --- | --- | --- | --- | --- | --- | --- |
|  | *CsABCG29* | TATGCCCGCTGCATTAAG | exon 20 | TGACACAGCTAAATTAGGAATGTGATA | UTR3’ | 150 | 60 |
|  | *CsABCG30* | TACTCCACATACCTATAGTTCCAA | UTR5’ | TTATTAATTCTGTGCCATGGAAGTTT | UTR5’/  exon 1 | 150 | 60 |
|  | *CsABCG31* | TCTTCGTCCTCCGCAAA | UTR5’ | GGATGAGCGAGAGCTTT | exon 1 | 200 | 60 |
|  | *CsABCG32* | CTCGGTAGCTATACATGACG | exon 24 | TTCCAAAGGAGATGAGAGGT | exon24/  UTR3’ | 156 | 56 |
|  | *CsABCG33* | CAAGAGATTAGAAGACTACAACGTAT | UTR5’ | ATCTTCAACGTCACTACCATT | exon 1 | 150 | 56 |
|  | *CsABCG34* | TAATTCTCCGTTGACACCG | UTR5’ | GGTAGCTGATGATCTAGCGA | exon 1 | 171 | 56 |
|  | *CsABCG35* | CCAAACCACAGCTTCAAGTA | exon 13 | ATATAATATTTAACGGCTTCTCTGAGCTA | exon 13 | 158 | 60 |
|  | *CsABCG37* | GAGGAGAGTGAGCCAGT | UTR5’ | GATAGACAGATTGCCGACG | exon 1 | 156 | 62 |
|  | *CsABCG38* | ATATCAGTACTTGTGGATGGTT | exon24 | ACATTATATGAGAGTGACAAGATTGAT | UTR3’ | 158 | 56 |
|  | *CsABCG39* | GGTTCAAATTCAAACTAAGAACCC | UTR5’ | GGGAGATATGGAATGCGC | exon 1 | 181 | 56 |
|  | *CsABCG41* | GGTGTTTAAAGAAACAACAACAATATCA | exon 22 | CAGAGAAGGTAAACACGTAAACTA | exon 22/  UTR3’ | 175 | 56 |
|  | *CsABCG42* | AAACAGTGGAAGAATTTGTGAG | exon 26 | GGGAATTGTGTATAGGCTTGTAT | UTR3’ | 192 | 56 |
|  | *CsABCG43* | CCCTCTGAAACTTATTGTGGT | UTR5’ | TAGACGCTTAAGAACAAGTTTATTGA | exon 1 | 166 | 56 |
|  | *CsABCG44* | TTTCCATTTCTCTCTACAAACATGA | UTR5’ | GTCGGCAACGGAAGAAG | exon 1 | 167 | 56 |
