# Supplementary material for: Genes Encoding Cucumber Full-Size ABCG Proteins Show Different Responses to Plant Growth Regulators and Sclareolide
Source: Plant Mol Biol Report. 2015 Nov 14;34:720–36. doi: 10.1007/s11105-015-0956-9 (PMC4923091; doi:10.1007/s11105-015-0956-9)
Supplement: Supplementary file 2 — (DOC 47 kb) [file 11105_2015_956_MOESM2_ESM.doc]

**Supplementary Table 2**

**RT-PCR conditions for organ expression analysis**

| **Gene *CsABCG*** | **Tm [ºC]** | **Number of cycles** | **Elongation time**  **[s]** | **EST accession number** |
| --- | --- | --- | --- | --- |
| *CsABCG29* (*CsPDR1*) | 60 | 30 | 60 | JZ719085 |
| *CsABCG30* (*CsPDR2*) | 60 | 30 | 60 | JZ714786 |
| *CsABCG31* (*CsPDR3*) | 60 | 30 | 60 | JZ719086 |
| *CsABCG32* (*CsPDR4*) | 56 | 30 | 60 | JZ714787 |
| *CsABCG33* (*CsPDR5*) | 56 | 30 | 60 | JZ714788 |
| *CsABCG34* (*CsPDR6*) | 56 | 30 | 60 | JZ714789 |
| *CsABCG35* (*CsPDR7*) | 60 | 30 | 60 | JZ719087 |
| *CsABCG37* (*CsPDR9*) | 62 | 34 | 60 | JZ714790 |
| *CsABCG38* (*CsPDR10*) | 56 | 30 | 60 | na^a^ |
| *CsABCG39* (*CsPDR11*) | 56 | 30 | 60 | JZ714791 |
| *CsABCG41* (*CsPDR13*) | 56 | 30 | 60 | JZ714792 |
| *CsABCG42* (*CsPDR14*) | 56 | 30 | 60 | JZ719088 |
| *CsABCG43* (*CsPDR15*) | 56 | 30 | 60 | JZ719089 |
| *CsABCG44* (*CsPDR16*) | 56 | 30 | 60 | JZ714793 |

^a^na - not available
